# Hereditary Variation in the Radial Arteries

**Published:** 1879-11

**Authors:** J. Schneck

**Affiliations:** Mt. Carmel, Ill.


### ARTICLE VII.

*Hereditary Variation in the Radial Arteries.*

The following diagram is the result of an examination of the radial arteries of Mr. J. W Brown and his family; and is such a remarkable example of hereditary transmission of an anatomical peculiarity that I cannot forbear asking space in the pages of the JOURNAL AND EXAMINER to publish it:

In the normal condition the radial artery passes along the inner side of the supinator longus muscle; on arriving at the wrist, "it winds backwards, round the outer side of the carpus, beneath the extensor tendons of the thumb;" but in the members of this family, consisting of grand-father, children and grand-children, 22 in all; in 15, one or both radial arteries take an abnormal course. Out of 44 arteries the same abnormal course is taken 19 times. Four times both arteries are abnormal, seven times both are normal. Twice the right only is abnormal, and nine times the left only is abnormal.

The deformity is similar in all the cases. The artery takes the usual course until within 3 to 4 centimeters of the wrist (according to the length of the arm), when it suddenly turns backwards over the supinator longus muscle, passing on the outside of the extensor tendons of the thumb and above the styloid process of the radius, thence behind the thumb into the palm, to form the palmer arch, etc. Grand-mother, daughters-in-law and sons-in-law all normal.

| Grand-father. | Children. | Grand-children. |
|---|---|---|
| G. W. Brown, Sr., both abnormal, and very crooked. | Mr. J. T. Brown, oldest child, left abnormal. | O. F. Brown, right abnormal.<br>A. P. Brown, both normal.<br>J. E. Brown, both abnormal.<br>M. E. Brown, both normal.<br>H. F. Brown, right abnormal. |
| | Mrs. Barnette, second child, left abnormal. | Willie Barnette, left abnormal.<br>Junia Barnette, left abnormal.<br>George Barnette, both abnormal.<br>Gertrude Barnette, left abnormal. |
| | Mrs. Ameter, third child, left abnormal. | Has no children. |
| | Mr. F. S. Brown, fourth child, left abnormal. | Has four children, all normal. |
| | Mrs. G. W. Brown, fifth child, left abnormal (married a man whose name is Brown.) | C. P. Brown, both abnormal.<br>M. F. Brown, both normal. |
| | Mr. G. W. Brown, jr., sixth child, left abnormal. | Has no children. |

Observe that in the "children" the left artery only is abnormal. Observe also in the family of Mr. F. S. Brown, who has four children, but none have the abnormality, in his own case the left is abnormal.

J. Schneck, m.d., Mt. Carmel, Ill.

